# Prediction model for cardiovascular disease in patients with diabetes using machine learning derived and validated in two independent Korean cohorts

**DOI:** 10.1038/s41598-024-63798-y

**Published:** 2024-06-28

**Authors:** Hyunji Sang, Hojae Lee, Myeongcheol Lee, Jaeyu Park, Sunyoung Kim, Ho Geol Woo, Masoud Rahmati, Ai Koyanagi, Lee Smith, Sihoon Lee, You-Cheol Hwang, Tae Sun Park, Hyunjung Lim, Dong Keon Yon, Sang Youl Rhee

**Affiliations:** 1 Department of Endocrinology and Metabolism, Kyung Hee University Medical Center, Kyung Hee University College of Medicine, 23 Kyungheedae-ro, Dongdaemun-gu, Seoul, 02447 South Korea; 2 Center for Digital Health, Medical Science Research Institute, Kyung Hee University Medical Center, Kyung Hee University College of Medicine, Seoul, South Korea; 3 Department of Family Medicine, Kyung Hee University Medical Center, Kyung Hee University College of Medicine, Seoul, South Korea; 4 Department of Neurology, Kyung Hee University Medical Center, Kyung Hee University College of Medicine, Seoul, South Korea; 5 Research Centre on Health Services and Quality of Life, Aix Marseille University, Marseille, France; 6 Department of Physical Education and Sport Sciences, Faculty of Literature and Human Sciences, Lorestan University, Khoramabad, Iran; 7 Department of Physical Education and Sport Sciences, Faculty of Literature and Humanities, Vali-E-Asr University of Rafsanjan, Rafsanjan, Iran; 8 Research and Development Unit, Parc Sanitari Sant Joan de Deu, Barcelona, Spain; 9 Centre for Health, Performance and Wellbeing, Anglia Ruskin University, Cambridge, UK; 10 Department of Internal Medicine, Gachon University College of Medicine, Incheon, South Korea; 11 Division of Endocrinology and Metabolism, Department of Internal Medicine, Kyung Hee University Hospital at Gangdong and Kyung Hee University School of Medicine, Seoul, South Korea; 12 Division of Endocrinology and Metabolism, Department of Internal Medicine, Research Institute of Clinical Medicine of Jeonbuk National University and Jeonbuk National University Hospital, Jeonju, South Korea; 13 Department of Medical Nutrition, Graduate School of East-West Medical Science, Kyung Hee University, Yongin, South Korea; 14 Department of Pediatrics, Kyung Hee University College of Medicine, 23 Kyungheedae-ro, Dongdaemun-gu, Seoul, 02447 South Korea; 15 Department of Regulatory Science, Kyung Hee University, Seoul, South Korea

**Keywords:** Machine learning, Cardiovascular diseases, Diabetes mellitus, Prediction, Random forest model, Diabetes complications, Disease prevention

## Abstract

This study aimed to develop and validate a machine learning (ML) model tailored to the Korean population with type 2 diabetes mellitus (T2DM) to provide a superior method for predicting the development of cardiovascular disease (CVD), a major chronic complication in these patients. We used data from two cohorts, namely the discovery (one hospital; n = 12,809) and validation (two hospitals; n = 2019) cohorts, recruited between 2008 and 2022. The outcome of interest was the presence or absence of CVD at 3 years. We selected various ML-based models with hyperparameter tuning in the discovery cohort and performed area under the receiver operating characteristic curve (AUROC) analysis in the validation cohort. CVD was observed in 1238 (10.2%) patients in the discovery cohort. The random forest (RF) model exhibited the best overall performance among the models, with an AUROC of 0.830 (95% confidence interval [CI] 0.818–0.842) in the discovery dataset and 0.722 (95% CI 0.660–0.783) in the validation dataset. Creatinine and glycated hemoglobin levels were the most influential factors in the RF model. This study introduces a pioneering ML-based model for predicting CVD in Korean patients with T2DM, outperforming existing prediction tools and providing a groundbreaking approach for early personalized preventive medicine.

## Introduction

Cardiovascular disease (CVD) and type 2 diabetes mellitus (T2DM) are intertwined health challenges that have profound implications for global morbidity and mortality^1^. T2DM is recognized as a risk factor for cardiovascular complications, contributing to an increased disease burden and adverse outcomes^1^. According to the 2022 Diabetes Fact Sheet in Korea, patients with prediabetes and diabetes have 1.05 and 1.59 times higher risks of myocardial infarction and 1.05 and 1.51 times higher risks of heart failure, respectively, compared to those in normoglycemic adults^2^. Preventive interventions for T2DM can prevent long-term complications and are reportedly cost-effective^3,4^. Cardiovascular risk factors known to date include duration of diabetes, obesity/overweight status, hypertension, dyslipidemia, smoking, a family history of early coronary artery disease, chronic kidney disease, and albuminuria^3,4^. All patients with diabetes should undergo annual assessments and management to identify modifiable abnormal risk factors^5^.

With the recent development of artificial intelligence, new technologies, such as machine learning (ML), have been applied in various fields. ML has been applied to texture filtering in computer graphics and images^6^ and rolling element-bearing fault identification in mechanical engineering. It is attracting attention as a new approach that overcomes the limitations of traditional methods and shows superior performance^6,7^. ML is gaining traction in the medical field, and efforts are being made to apply ML technologies to the existing disease models^8,9^. A study that used an ML algorithm to predict recurrent spontaneous abortion constructed a practical framework using clinical information from real clinical data, such as vitamin D and thyroid function tests, in the analysis and achieved a sensitivity of 93.3% and specificity of 93.1%^10^. The study involving a novel transfer learning-based ensemble classifier for detecting COVID-19 infection in chest computed tomography scans provides an important diagnostic tool for the field of medical imaging and is expected to have a significant impact on the diagnosis and management of various other lung diseases in the future^11^. ToxMVA, a deep learning-based method used for predicting protein toxicity in the early stages of protein-based drug discovery, has shown the potential to shorten the process and reduce the cost of drug screening^12^. Thus, ML is a powerful tool for addressing existing limitations by leveraging clinical data to uncover hidden patterns and identifying critical variables associated with disease development. By integrating ML algorithms with clinical data, clinicians can efficiently identify early warning signs and risk factors for complications^13^.

Various methods have been developed to assess and predict CVD risk, including atherosclerotic CVD risk calculators and biomarkers^14,15^. However, this is problematic because traditional risk assessment tools are insufficient and the complex interactions between clinical variables are unclear^8,9^. Various predictive models using ML have recently been developed for CVD prediction in T2DM; however, their predictive power is limited because multiple risk factors have not been included in these models^16–18^. Therefore, this study aimed to identify the relationship between clinical factors and cardiovascular complications and to develop a predictive model for CVD occurrence through rigorous model training and validation, utilizing the strengths of ML technology in patients with T2DM in South Korea. By identifying significant predictors within the model, we sought to understand the nuanced relationship between these factors and cardiovascular risk in patients with T2DM. We have presented our methodology, including a thorough validation process for two independent Korean cohorts, strengthening the reliability and applicability of our findings. We have also discussed the potential of the predictive model developed in this study, emphasizing its role as an efficient and accurate tool for CVD risk stratification and its contribution to the field of personalized medicine to improve CVD outcomes.

## Materials and methods

### Study population and data collection

**Figure 1:**
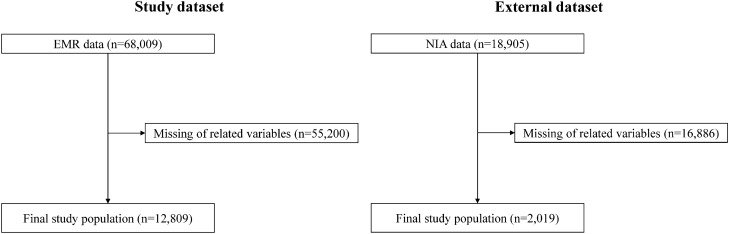
Study workflow.

The data used in this retrospective study were obtained from two independent longitudinal cohorts previously enrolled in an observational study. Hospital-based data consisted of electronic medical records from outpatient, inpatient, and emergency department visits collected between January 1, 2008, and December 31, 2022. Eligible participants were selected from among patients with T2DM, excluding those with type 1 diabetes and previous onset of CVD. Finally, 12,809 patients were selected from a tertiary hospital at the Kyung Hee University Medical Center for the discovery cohort. Data for extra-validation were collected from a retrospective dataset from the secondary hospitals Kyung Hee University Medical Center at Gangdong and Gachon University Gil Hospital (validation cohort), and 2019 eligible patients were selected (Fig. 1).

### Input variables

A comprehensive set of 68 variables was included in the model. Patients’ baseline demographic characteristics included age and sex. Medical histories included the presence of hypertension, dyslipidemia, macrovascular complications (cerebrovascular disease, dementia, Parkinson’s disease, and lower limb amputation), microvascular complications (diabetic retinopathy, proliferative diabetic retinopathy, diabetic neuropathy, and chronic kidney disease), and cancer. Medication history included types of antidiabetic drugs (metformin, sulfonylurea, dipeptidyl peptidase-4 inhibitor, meglitinide, thiazolidinedione, α-glucosidase inhibitor, insulin, glucagon-like peptide-1 receptor agonist, and sodium–glucose co-transporter 2 inhibitor), antihypertensive drugs (angiotensin II receptor blocker, angiotensin-converting enzyme inhibitor, calcium channel blocker [CCB], diuretics, and beta blocker), dyslipidemia drugs (statin, fibrate, ezetimibe, omega-3, and other dyslipidemia drugs), and antiplatelet agents (aspirin, clopidogrel, cilostazol, glycoprotein IIb/IIIa antagonist, and other antiplatelet agents). The mean and range of clinical parameters included body mass index (BMI)^19^, systolic blood pressure, diastolic blood pressure, and pulse rate. The mean and range of blood tests included glycated hemoglobin (HbA1c), serum glucose, total cholesterol, triglycerides, high-density lipoprotein cholesterol, low-density lipoprotein (LDL) cholesterol, serum creatinine, aspartate aminotransferase (AST), alanine aminotransferase (ALT), gamma-glutamyl transferase, and alkaline phosphatase (ALP) levels.

### Identification of new CVD cases

New-onset CVD among patients with T2DM was identified using the International Classification of Diseases, 10th Revision (ICD-10) codes for ischemic heart disease and myocardial infarction (I20.X–I25.X), heart failure (I50.X), and atrial fibrillation (I48.X). The primary endpoint was a new CVD diagnosis within 3 years.

### Data preprocessing

Missing data were excluded from analysis. Covariates were divided into three sections: (1) demographic data, (2) physical examinations and blood tests, and (3) medication and comorbidity information. Using the examination date, we utilized physical examination and blood test data before the CVD onset. The dataset for the entire period was calculated and converted into a mean. During this period, the range was calculated by subtracting the maximum and minimum recorded values. First-visit information on medications and comorbidities was selected as a covariate.

### Model training and validation

A common ML approach for prediction involves splitting data into training and test sets. In this study, the target value of the given data on the incidence of CVD over 3 years was insufficient. Consequently, the model was trained on the entire dataset, rather than being split for internal validation. Instead, a separate external dataset was used to assess the extent to which the model could be generalized. This approach is essential to verify whether the model performs well on previously unseen data.

### Model development

We selected decision-tree-based ensemble models, such as the XGBoost (XGB), random forest (RF), LightGBM (LGM), and AdaBoost (ADB), and linear classification models, such as logistic regression (LR) and support vector machine (SVM). Among these, the XGB, RF, and LGM models are the most common and practical models for handling a mixture of categorical and continuous variables. For the SVM model, we chose a linear kernel because of its simplicity and efficiency, particularly for high-dimensional data, where the number of features is much larger than the number of samples. Linear kernels are beneficial when data are linearly or nearly linearly separable.

To optimize the performance of each model, we performed hyperparameter tuning using GridSearchCV and maximized the area under the receiver operating characteristic curve (AUROC) to determine the best combination of hyperparameters.

### ML analysis

To determine the AUROC score, various tree-based and linear classification models were used to predict the potential occurrence of CVD. Using the AUROC score as a scoring metric, we used GridSearchCV to optimize the hyperparameters of this model. Once the optimal hyperparameters were determined, the model was trained for subsequent predictions. Considering the class imbalance in our data, we used the synthetic minority over-sampling technique to generate synthetic samples.

We used various metrics, such as AUROC, accuracy, sensitivity, specificity, and balanced accuracy, to assess the model's performance. These metrics were calculated based on the probability predictions generated by the model. Subsequently, we calculated the mean and 95% confidence intervals (CIs) for each performance metric, measuring both the average and variability of the model's performance. Due to characteristics such as the small size of the external dataset, we used a bootstrapping method instead of traditional cross-validation. By resampling the dataset multiple times and performing bootstrapping with up to 10,000 iterations, we could evaluate the sample distribution and calculate 95% CIs for the model's performance metrics. We plotted a receiver operating characteristic (ROC) curve to visually represent the model’s performance. This was complemented by the mean ROC curve and the standard deviation within that range, which elucidated the distribution of the model's performance.

To identify the most important features for predicting CVD, we utilized the intrinsic feature-importance mechanisms provided by tree-based models such as the RF, XGB, and LGM, which primarily evaluate the feature-importance based on metrics such as mean decrease impurity method, which calculates Gini impurity reduction and gain. We selected the top 15 features that had the greatest impact on the model and plotted them on a bar graph to illustrate their influence on the model predictions.

### Performance metrics

To understand the performance of our model comprehensively, we selected the following five performance metrics: AUROC, accuracy, sensitivity, specificity, and balanced accuracy. AUROC is a robust performance measure to a model's ability to discriminate between classes across all possible thresholds. Its robustness stems from the fact that it considers both sensitivity and specificity, making it the preferred metric, particularly in situations where classes are unbalanced. The accuracy is a simple and intuitive performance metric that provides the proportion of true results (both true positives and true negatives) from the total number of cases examined. However, accuracy alone can be misleading, particularly for unbalanced datasets; therefore, additional performance metrics are required. Sensitivity and specificity were selected to assess how well the model identified the positive and negative cases, respectively. Sensitivity measures the proportion of true positives correctly identified by the model and provides insight into the model's ability to detect positive cases, whereas specificity measures the proportion of actual negatives that are correctly identified, providing a sense of the model's ability to avoid false alarms. Finally, we included balanced accuracy to provide a more balanced view of our model's performance, particularly in the face of class imbalance. As an average of the sensitivity and specificity, balanced accuracy assigns equal weights to both metrics, making it a good alternative to accuracy when addressing unbalanced datasets. The combination of these metrics enabled us to evaluate the performance of our model from different perspectives, thereby ensuring a more robust evaluation^20,21^.

### Software and libraries

All data preprocessing, model development, and analyses were performed using Python 3.9.16. Key libraries used in our study included Scikit-learn 1.2.2, NumPy 1.23.5, and Pandas 1.5.3 for ML algorithms and data manipulation. Matplotlib 3.7.1 and Seaborn 0.12.2 were used for data visualization.

#### Ethical approval

This study was approved by the Institutional Review Board of the Kyung Hee University Hospital (No. KHSIRB-22–473(EA)). The requirement for informed consent was waived by the institutional review board because deidentified data were used for the analyses. All research was performed in accordance with the relevant guidelines, regulations, and the Declaration of Helsinki. This study followed the guidelines outlined in the Transparent Reporting of a Multivariate Prediction Model for Individual Prognosis or diagnosis (TRIPOD) statement^22^.

## Results

### Cohort characteristics

In total, 12,809 patients were selected from the discovery cohort, of whom 1238 (10.2%) had CVD (Fig. 1). Among the participants, 6530 (51.0%) were male patients, and the mean age was 62.5 ± 12.1 years. For extra-validation, 2019 patients were included, comprising 32 (1.6%) patients with CVD from the validation cohort. The validation cohort had 1094 (54.2%) male patients with a mean age of 56.3 ± 11.9 years (Table 1). The median range of each clinical indicator and blood test was the difference between the maximum and minimum values before the occurrence of CVD in the hospital records of each patient, listed separately in Supplementary Table S1.

**Table 1:** Baseline characteristics of the study and extra-validation datasets.

	Study dataset			Extra-validation dataset		
	Total	Control	Case^a^	Total	Control	Case^a^
Number, N	12,809	11,571	1238	2019	1966	32
Age	62.5 (12.05)	62.1 (12.15)	66.0 (10.46)	56.3 (11.94)	56.2 (11.92)	60.5 (12.11)
Male sex, N (%)	6530 (50.98)	5859 (50.64)	671 (54.20)	1094 (54.19)	1058 (53.81)	36 (67.92)
Body mass index, kg/m^2^	24.9 (3.75)	24.9 (3.74)	25.4 (3.78)	25.2 (3.62)	25.2 (3.64)	24.5 (3.11)
Systolic BP, mmHg	124.1 (14.30)	124.2 (14.24)	123.4 (14.82)	128.0 (13.36)	128.0 (13.27)	126.1 (16.30)
Diastolic BP, mmHg	75.7 (8.96)	75.8 (8.91)	74.3 (9.34)	76.4 (8.54)	76.4 (8.53)	75.7 (9.13)
Pulse rate, beats/min	75.6 (10.62)	75.9 (10.54)	73.6 (11.13)	79.9 (10.63)	79.9 (10.59)	78.7 (12.01)
HbA1c, %	6.9 (1.01)	6.9 (1.00)	6.9 (1.03)	7.2 (1.09)	7.2 (1.08)	7.5 (1.09)
Fasting blood glucose, mg/dL	151.3 (46.35)	151.6 (45.87)	148.9 (50.49)	146.7 (46.10)	146.5 (46.21)	155.9 (41.31)
Total cholesterol, mg/dL	160.7 (32.86)	160.7 (32.42)	160.4 (36.70)	166.2 (32.09)	165.9 (31.84)	175.0 (39.65)
Triglyceride, mg/dL	143.8 (69.12)	143.3 (68.51)	148.7 (74.47)	147.2 (66.68)	146.6 (66.35)	169.5 (75.41)
HDL cholesterol, mg/dL	47.4 (12.37)	47.6 (12.30)	45.5 (12.83)	46.4 (10.82)	46.5 (10.83)	42.7 (9.85)
LDL cholesterol, mg/dL	93.0 (26.84)	92.9 (26.48)	93.8 (29.93)	94.6 (29.56)	94.5 (29.47)	99.5 (32.63)
Creatinine, mg/dL	0.9 (0.45)	0.9 (0.44)	0.9 (0.47)	1.0 (0.54)	1.0 (0.53)	1.2 (0.78)
AST, U/L	26.7 (13.42)	26.7 (13.55)	26.9 (12.11)	24.5 (8.36)	24.5 (8.36)	24.7 (8.38)
ALT, U/L	24.0 (13.62)	23.9 (13.38)	25.1 (15.66)	25.5 (12.83)	25.4 (12.77)	27.0 (15.14)
GGT, U/L	39.5 (37.36)	39.2 (37.41)	41.9 (36.79)	37.3 (30.38)	37.2 (30.39)	42.1 (29.68)
ALP, U/L	79.8 (25.50)	80.1 (25.52)	77.0 (25.20)	165.8 (79.31)	166.2 (79.17)	152.2 (84.11)
Co-morbid conditions						
Hypertension	6995 (54.61)	6188 (53.48)	807 (65.19)	894 (44.28)	867 (44.10)	27 (50.94)
Dyslipidemia	5435 (42.43)	4748 (41.03)	687 (55.49)	790 (39.13)	767 (39.01)	23 (43.40)
Macrovascular complications						
Cerebrovascular diseases	1317 (10.28)	1212 (10.47)	105 (8.48)	135 (6.69)	130 (6.61)	5 (9.43)
Dementia	207 (1.62)	190 (1.64)	17 (1.37)	18 (0.89)	18 (0.92)	0 (0.00)
Parkinson's disease	96 (0.75)	81 (0.70)	15 (1.21)	5 (0.25)	4 (0.20)	1 (1.89)
Peripheral vascular disease	150 (1.17)	136 (1.18)	14 (1.13)	133 (6.59)	125 (6.36)	8 (15.09)
Lower limb amputation	3 (0.02)	3 (0.03)	N/A	N/A	N/A	N/A
Microvascular complications						
Retinopathy	191 (1.49)	179 (1.55)	12 (0.97)	220 (10.90)	215 (10.94)	5 (9.43)
Proliferative diabetic retinopathy	247 (1.93)	231 (2.00)	16 (1.29)	53 (2.63)	53 (2.70)	N/A
Chronic kidney disease	356 (2.78)	319 (2.76)	37 (2.99)	263 (13.03)	252 (12.82)	11 (20.75)
Neuropathy	706 (5.51)	661 (5.71)	45 (3.63)	283 (14.02)	270 (13.73)	N/A
Cancer	685 (5.35)	649 (5.61)	36 (2.91)	103 (5.10)	100 (5.09)	3 (5.66)
*Medication use*						
Diabetes mellitus						
Metformin	4709 (36.76)	4263 (36.84)	446 (36.03)	734 (36.35)	719 (36.57)	15 (28.30)
Sulfonylurea	2017 (15.75)	1848 (15.97)	169 (13.65)	172 (8.52)	168 (8.55)	4 (7.55)
DPP-4 inhibitor	1713 (13.37)	1540 (13.31)	173 (13.97)	64 (3.17)	63 (3.20)	1 (1.89)
Meglitinide	90 (0.70)	86 (0.74)	4 (0.32)	34 (1.68)	32 (1.63)	2 (3.77)
Thiazolidinedione	190 (1.48)	179 (1.55)	11 (0.89)	2 (0.10)	2 (0.10)	N/A
α-Glucosidase inhibitor	105 (0.82)	103 (0.89)	2 (0.16)	28 (1.39)	28 (1.42)	N/A
Insulin	2017 (15.75)	1848 (15.97)	169 (13.65)	N/A	N/A	N/A
GLP-1 receptor agonist	3 (0.02)	3 (0.03)	N/A	N/A	N/A	N/A
SGLT2 inhibitor	104 (0.81)	86 (0.74)	18 (1.45)	N/A	N/A	N/A
Hypertension						
Angiotensin II receptor blocker	3391 (26.47)	3036 (26.24)	355 (28.68)	92 (4.56)	88 (4.48)	4 (7.55)
ACE inhibitor	137 (1.07)	118 (1.02)	19 (1.53)	19 (0.94)	18 (0.92)	1 (1.89)
Calcium channel blocker	3299 (25.76)	2982 (25.77)	317 (25.61)	228 (11.29)	224 (11.39)	4 (7.55)
Diuretics	1486 (11.60)	1348 (11.65)	138 (11.15)	41 (2.03)	39 (1.98)	2 (3.77)
Beta blocker	1120 (8.74)	881 (7.61)	239 (19.31)	1 (0.05)	1 (0.05)	N/A
Dyslipidemia						
Statin	4504 (35.16)	3897 (33.68)	607 (49.03)	478 (23.68)	463 (23.55)	15 (28.30)
Fibrate	111 (0.87)	105 (0.91)	6 (0.48)	52 (2.58)	50 (2.54)	2 (3.77)
Ezetimibe	231 (1.80)	174 (1.50)	57 (4.60)	35 (1.73)	34 (1.73)	1 (1.89)
Omega-3	173 (1.35)	153 (1.32)	20 (1.62)	18 (0.89)	18 (0.92)	N/A
ETC dyslipidemia^b^	6 (0.05)	5 (0.04)	1 (0.08)	13 (0.64)	13 (0.66)	N/A
Antiplatelet						
Aspirin	1756 (13.71)	1564 (13.52)	192 (15.51)	288 (14.26)	279 (14.19)	9 (16.98)
Clopidogrel	1382 (10.79)	1107 (9.57)	275 (22.21)	70 (3.47)	67 (3.41)	3 (5.66)
Cilostazol	796 (6.21)	730 (6.31)	66 (5.33)	60 (2.97)	58 (2.95)	2 (3.77)
Glycoprotein IIb/IIIa antagonist	19 (0.15)	19 (0.16)	N/A	N/A	N/A	N/A
ETC antiplatelet^b^	796 (6.21)	687 (5.94)	109 (8.80)	N/A	N/A	N/A

Data are presented as mean (standard deviation) or N (%) unless indicated otherwise.

*BP* blood pressure, *HbA1c* glycated hemoglobin, *HDL* high-density lipoprotein, *LDL* low-density lipoprotein, *AST* aspartate transaminase, *ALT* Alanine transaminase, *GGT* gamma-glutamyl transferase, *ALP* alkaline phosphatase, *DPP-4* dipeptidyl peptidase-4, *GLP-1* glucagon-like peptide-1, *SGLT2* sodium glucose cotransporter 2, *ACE* angiotensin-converting-enzyme.

^a^Group of patients with newly developed cardiovascular disease within 3 years.

^b^ETC stands for "et cetera" and is used here to mean "other types of".

### Comparisons of prediction model performance

**Figure 2:**
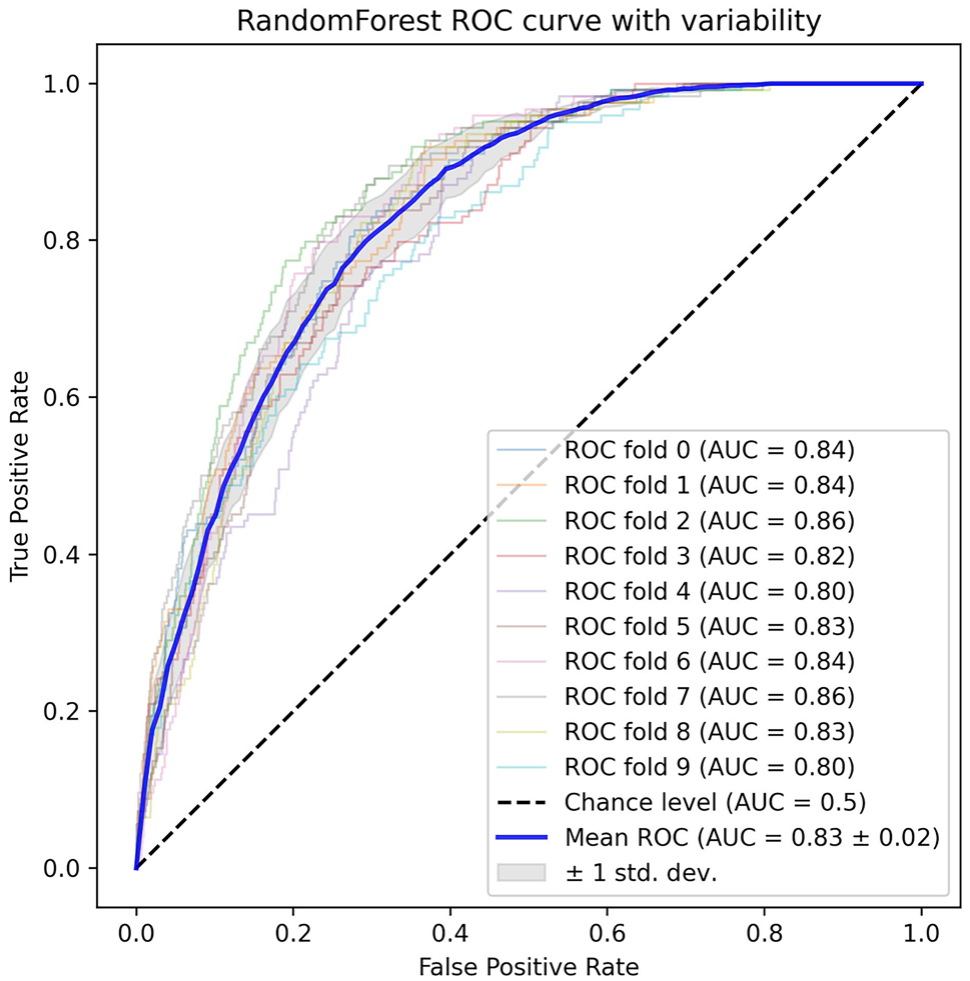
ROC curves of the random forest model. Mean ROC curve from tenfold cross-validation on the original dataset. *ROC* receiver operating characteristic, *AUC* area under the ROC curve.

The RF model displayed impressive performance on the validation set, exhibiting an AUROC of 0.830 (95% CI, 0.816–0.842). Details of the hyperparameter of the models and the tuning process of the RF model are listed in Supplementary Tables S2 and S3. The RF model also demonstrated consistent metrics across the board (accuracy: 74.7% [73.2–76.2]; sensitivity: 74.6% [73.0–76.2]; specificity: 74.7% [73.2–76.2]; and balanced accuracy: 74.6% [73.1–76.2]). In comparison, the XGB and LGM models demonstrated slightly higher values of AUROC and maintained an overall robust performance. The ADB and LR models presented consistent performance despite a lower AUROC than that of the RF model. The SVM model delivered a substantially lower AUROC value and demonstrated noticeable variability in its metrics (Table 2; Fig. 2).

**Table 2:** Performance metrics of six different machine learning algorithms on the original and external validation datasets.

Model	AUROC	Accuracy, %	Sensitivity, %	Specificity, %	Balanced accuracy, %
Validation results, mean (95% CI)					
XGB	0.845 (0.829–0.861)	76.1 (74.5–77.7)	76.0 (74.5–77.6)	76.1 (74.5–77.7)	76.1 (74.5–77.6)
RF	0.830 (0.818–0.842)	74.7 (73.2–76.2)	74.6 (73.0–76.2)	74.7 (73.2–76.2)	74.6 (73.1–76.2)
LGM	0.842 (0.826–0.858)	75.9 (73.9–77.9)	75.8 (73.9–77.7)	75.9 (73.9–77.9)	75.9 (73.9–77.8)
ADB	0.817 (0.800–0.835)	73.9 (72.1–75.6)	74.1 (72.2–76.0)	73.8 (72.1–75.6)	74.0 (72.1–75.8)
LR	0.786 (0.767–0.806)	71.9 (69.8–74.0)	71.9 (69.8–74.0)	71.9 (69.8–74.0)	71.9 (69.8–74.0)
SVM	0.488 (0.411–0.565)	42.0 (29.7–54.3)	58.6 (43.5–73.7)	40.2 (25.4–55.0)	49.4 (44.1–54.7)
External results					
XGB	0.710 (0.649–0.770)	64.7 (59.4–71.8)	64.6 (58.7–72.3)	64.7 (59.4–71.8)	64.6 (59.2–72.0)
RF	0.722 (0.660–0.783)	66.4 (62.2–72.0)	66.4 (60.9–72.6)	66.4 (62.2–72.0)	66.4 (61.7–72.2)
LGM	0.717 (0.653–0.778)	64.9 (58.0–73.1)	64.9 (57.6–73.1)	64.9 (58.1–73.1)	64.9 (57.9–73.2)
ADB	0.716 (0.654–0.779)	65.3 (59.2–71.1)	65.3 (59.0–71.0)	65.3 (59.2–70.7)	65.3 (59.2–70.6)
LR	0.717 (0.636–0.791)	65.4 (59.6–70.7)	65.4 (59.6–70.7)	65.4 (59.6–70.7)	65.4 (59.6–70.6)
SVM	0.526 (0.449–0.604)	50.8 (44.9–56.6)	50.8 (44.4–56.9)	50.8 (44.9–56.6)	50.8 (44.9–56.6)

The performance metrics of six machine learning models (XGBoost, random forest, LightGBM, AdaBoost, Logistic Regression, and SVM) were used to predict the onset of cardiovascular disease within 3 years in patients using both the original and additional external validation datasets.

*AUROC* area under the receiver operating characteristic curve, *XGB* XGBoost, *RF* random forest, *LGM* LightGBM, *ADB* AdaBoost, *LR* logistic regression, *SVM* support vector machine.

When these models were applied to the external validation set, the RF model achieved the highest AUROC of 0.722 and exhibited the best performance on the other metrics. The XGB, LGM, ADB, and LR models exhibited lower performance than did the RF model and demonstrated good results for other performance metrics, whereas the SVM model exhibited somewhat less efficacy (Table 2; Fig. 2).

Consequently, considering its superior and consistent results, the RF model emerged as the most effective predictor of CVD onset within a 3-year timeframe in patients with diabetes.

### Contributing factors for the prediction model performance

**Figure 3:**
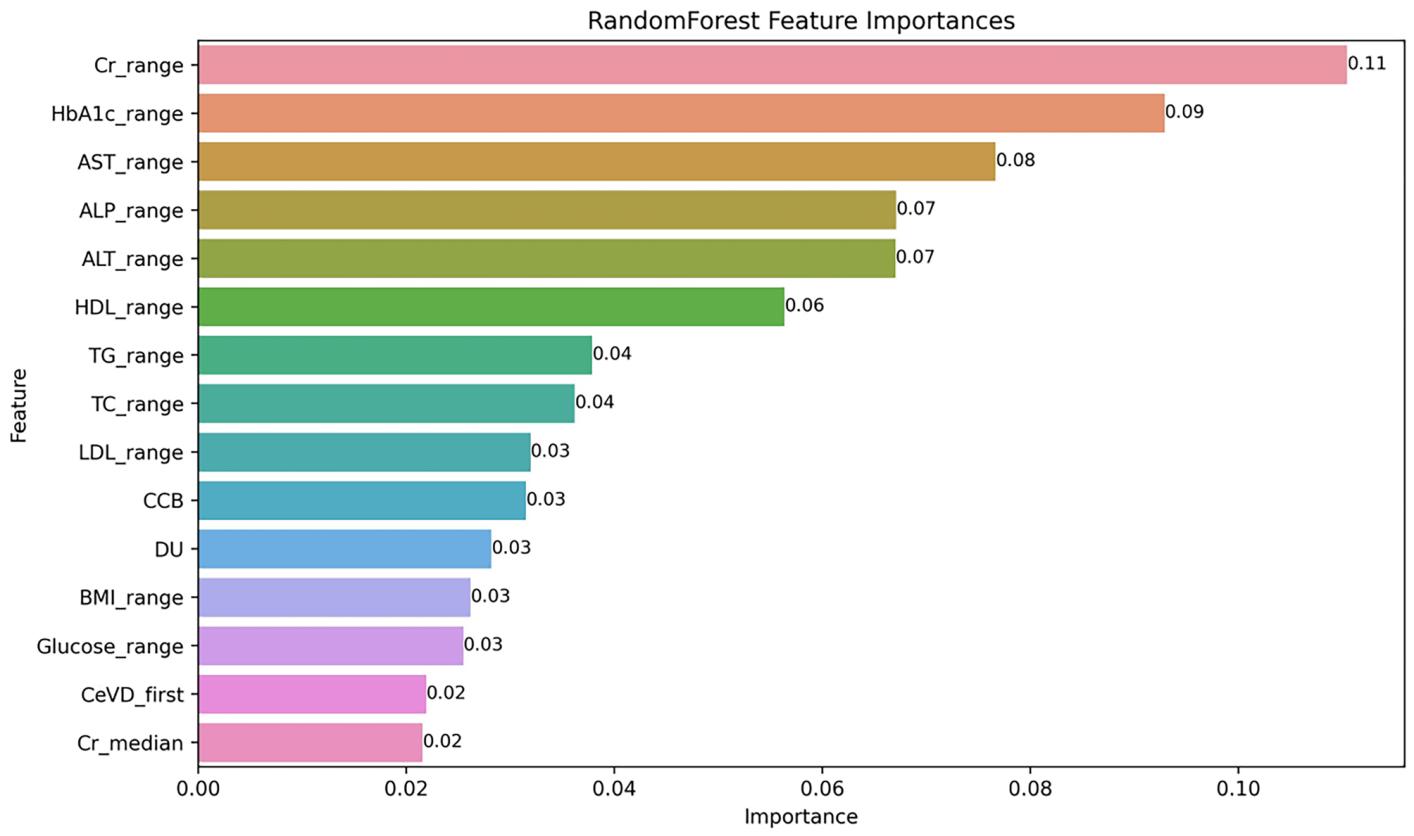
Top 15 feature-importance of the random forest model. *Cr* creatinine, *HbA1c* glycated hemoglobin, *AST* aspartate transaminase, *ALP* alkaline phosphatase, *ALT* alanine transaminase, *HDL* high-density lipoprotein, *TG* triglyceride, *TC* total cholesterol, *LDL* low-density lipoprotein, *CCB* calcium channel blocker, *DU* diuretics, *BMI* body mass index, *CeVD* cerebrovascular disease.

The significance of the contributing factors analyzed using the feature-importance method is shown in Fig. 3. Among the 68 variables considered in this study, the most critical factor contributing to the performance of the CVD prediction model was creatinine level, followed by HbA1c, AST, ALP, and ALT levels. BMI and medication history of CCB and diuretics were also included among the top 15 features. Early cerebrovascular complications were among the top 15 features. To provide an insight into the performance of the RF model, we have visually presented the distribution of each of the top 15 most important features in Supplementary Fig. S1.

## Discussion

This study highlights the importance of developing a highly accurate ML-based CVD prediction model that can be universally applied to adults with T2DM in South Korea, facilitating easy and accurate assessment of future annual CVD risk in the diabetic population. To summarize the results of this study, the XGB, RF, LGM, and ADB models, which are ensemble models, demonstrated excellent performance with AUROC values of 0.81–0.84 on the internal validation dataset and 0.71–0.72 on the external validation dataset. Creatinine and HbA1c levels ranked highest among the top 15 feature-importance factors (Fig. 3). The findings of this study can potentially improve patient outcomes by facilitating timely interventions, enhancing the understanding of contributing variables, and reducing the burden of cardiovascular complications in patients with diabetes.

The pooled cohort equation and Framingham risk score, which are standard prognostic models based on classical risk factors, were developed and performed well in predicting CVD in the general population^23^, but they failed to provide reliable prediction results in patients with diabetes^24,25^. Specifically, the Framingham prediction model was validated three times in a population with diabetes; however, the area under the curve (AUC) varied widely between 0.56 and 0.80 and was poorly calibrated (*P* < 0.001)^26^. Many ML models have been developed to predict incident CVD in the general population; however, their usefulness remains unclear owing to methodological flaws, a lack of external validation, and model impact studies^27^. Various ML models for predicting cardiovascular risk in patients with T2DM have not been extensively studied. According to a systematic review, the neural network-based model performed the best with an AUC of 0.91; however, the precision of the model was only 76.6% and no external validation was not performed^28^. In contrast, our CVD prediction model demonstrated sufficiently good performance with a mean AUROC of 0.83, using only questionnaires, body measurements, and blood tests commonly conducted in clinical practice for patients with diabetes. In addition, our prediction model maintained an excellent performance even after external validation.

This study differs from previous studies in that it was based on a large cohort of Koreans, utilizing data from three university hospitals and showed a superior predictive performance compared to the existing risk prediction models. The discovery cohort from one university hospital used for model training and the cohorts from the two university hospitals used for validation comprised different populations with different baseline characteristics. The performance of the model developed in this study trained on one cohort was similar to that of independent cohorts with different characteristics. We utilized the RF model to analyze CVD risk factors, including medical history, clinical parameters, and blood tests, due to its ability to handle multicollinearity among variables such as blood pressure, glycemic status, and dyslipidemia. This model choice enhances the stability and reliability of our results, allowing us to effectively use interrelated predictors without the risks associated with multicollinearity in linear models^29–31^. Despite the RF model's robustness in handling multicollinearity, interpreting feature-importances requires careful consideration of their potential redundancy and the nuanced relationship between mathematical and clinical significance. In addition, this study used the range of each clinical variable and blood test results as input variables. Among the top 15 features, 11 involved range values rather than the median values of each variable. This suggests that the fluctuation of these variables is more critical in risk prediction than the baseline values of the variables, which are considered classic risk factors in the existing risk prediction models.

Chronic kidney disease is also a known risk factor for CVD^32^, and predicting the occurrence of CVD using albuminuria, estimated glomerular filtration rate (eGFR), or cystatin C is feasible^33^. Among ML-based CVD prediction models, one includes creatinine levels^34^. A previous study also indicated that eGFR variability predicts cardiovascular event-induced hospitalization and death better than the baseline eGFR^35^. Consistent with these findings, our study demonstrated that the change in creatinine levels was more critical in predicting CVD in patients with diabetes than the median value of baseline creatinine.

Persistently elevated high blood glucose levels cause a hyperglycemic burden and adversely affect the occurrence of complications. Recent studies have reported that HbA1c variability plays an important role in microvascular disease outcomes in patients with relatively optimal basal glycemic control and that high HbA1c variability can predict almost all cardiovascular complications of T2DM^36,37^. Thus, it is reasonable to explain why the range of change in HbA1c holds importance in the CVD prediction model used in this study. Even if the median HbA1c level is low, high glycemic variability increases the risk of CVD complications.

Moreover, the finding that variability in liver levels and lipid profiles can be a predictor of CVD in this study is supported by previous studies^37–39^. However, conflicting results have been reported regarding the effects of BMI and LDL cholesterol variability on CVD^40,41^. Furthermore, among the complications other than cardiovascular complications in diabetes, the presence or absence of cerebrovascular disease is an important variable for predicting CVD risk. Previous studies reporting that the total area of carotid plaques predicts the risk of myocardial infarction support this result^42^. However, it is difficult to explain why cerebrovascular complications are more important predictors than microvascular or macrovascular complications. CCB and diuretics are the most commonly used medications for treating hypertension. Diuretics are among the most widely used drugs for triple therapy in patients with uncontrolled hypertension. This disproves the idea that uncontrolled hypertension may increase the risk of CVD, although the median or range of systolic or diastolic blood pressure was not included among the top 15 features.

This study has several limitations. First, owing to the retrospective nature of the study, it was challenging to expect detailed precision in the information obtained from the dataset based on hospital medical records. Information bias, arising from inaccuracies in data collection methods and the recording of medications and clinical parameters, can lead to misclassification of both exposure and outcome, affecting the accuracy of an ML model's predictions for CVD in patients with T2DM. Identification of new-onset CVD cases by ICD-10 codes is limited by potential misclassification and failure to capture patient nuances, and comprehensive record checks are required to validate “negative” CVD cases as defined by ICD-10 codes. Nevertheless, the ICD-10 codes provide a standardized methodology that is important for large-population studies. Second, the model was trained on data obtained from tertiary care centers, which may introduce a selection bias because these patients may have socioeconomic backgrounds different from those of primary care patients and receive more comprehensive healthcare, affecting CVD risk and diabetes management. Third, the superiority of this prediction model has not been compared with other existing prediction models and warrants further research. Finally, this study alone could not prove a causal relationship between the predictors used in the model and the occurrence of CVD. Confounding factors in predicting CVD in patients with T2DM include possible biases due to medications prescribed for conditions such as CVD risk factors (hypertension or dyslipidemia), the influence of unexplained variables such as the severity and duration of diabetes, and missing data on important lifestyle factors (smoking status, physical activity, diet, and alcohol consumption), all of which can distort the true relationship between risk factors and CVD outcomes.

Nevertheless, this study used an ML-based CVD prediction model for patients with T2DM using comprehensive covariates and two independent cohorts from a multicenter registry in South Korea. In the future, it may be possible to leverage common data models such as the Observational Medical Outcome Partners-Common Data Model, which integrates data from multiple institutions worldwide to create more accurate and precise global ML-based CVD prediction models.

## Conclusion

We successfully constructed an ML-based predictive model using a representative national cohort, enabling easy and accurate prediction of CVD risk in all members of the Korean population with T2DM. Our model outperformed traditional risk assessment tools, such as the Framingham risk score, which has shown limitations in their applicability to patients with diabetes. Highlighting the importance of creatinine and HbA1c level variabilities, the study illustrates how well-developed ML models can predict CVD risk across diverse populations, using routine clinical data to enhance risk assessment accessibility. Although much work remains to be done to generalize our model to a more diverse diabetic population, as it was trained on a specific cohort, we aimed to show that the ML model we developed and validated has broad potential for predicting CVD events in a diverse population of patients with diabetes. As part of a national project with the Korea Disease Control and Prevention Agency and the Korean Diabetes Association, this pioneering implementation prompts further expansion and validation of these models to diverse settings, reinforcing ML's role in advancing healthcare outcomes. Despite the conservative stance of several national clinical guidelines for diabetes and CVD on the use of predictive models, our findings support a reassessment of the clinical integration of these models to improve personalized medical approaches and reduce the CVD burden. Future research should focus on improving these models by including a broader range of patient data and conducting comparative studies using existing prediction methods. Collaboration between healthcare professionals, data scientists, and policymakers is essential to update clinical guidelines and ensure ethical use, paving the way for improved patient outcomes and the advancement of personalized medicine in public health.

### Supplementary Information

Supplementary Information.

## Data Availability

The datasets generated and/or analyzed during the current study are not publicly available owing to patient confidentiality or because they are used under license but are available from the corresponding author upon reasonable request.
