# Supplementary material for: Prediction model for cardiovascular disease in patients with diabetes using machine learning derived and validated in two independent Korean cohorts
Source: Sci Rep. 2024 Jun 28;14:14966. doi: 10.1038/s41598-024-63798-y (PMC11213851; doi:10.1038/s41598-024-63798-y)

Original Article

Prediction Model for Cardiovascular Disease in Patients with Diabetes Using Machine Learning Derived and Validated in Two Independent Korean Cohorts

Running title: Machine learning model to predict diabetic CVD

Hyunji Sang[1,2,†], Hojae Lee[2,†], Myeongcheol Lee[2], Jaeyu Park[2], Sunyoung Kim[3], Ho Geol Woo[4], Masoud Rahmati[5,6,7], Ai Koyanagi[8], Lee Smith[9], Sihoon Lee[10], You-Cheol Hwang[11], Tae Sun Park[12], Hyunjung Lim[13], Dong Keon Yon[2,14,*], Sang Youl Rhee[1,2,15,*]

[1]Department of Endocrinology and Metabolism, Kyung Hee University Medical Center, Seoul, South Korea

[2]Center for Digital Health, Medical Science Research Institute, Kyung Hee University Medical Center, Kyung Hee University College of Medicine, Seoul, South Korea

[3]Department of Family Medicine, Kyung Hee University Medical Center, Kyung Hee University College of Medicine, Seoul, South Korea

[4]Department of Neurology, Kyung Hee University Medical Center, Kyung Hee University College of Medicine, Seoul, South Korea

[5]Research Centre on Health Services and Quality of Life, Aix Marseille University, Marseille, France.

[6]Department of Physical Education and Sport Sciences, Faculty of Literature and Human Sciences, Lorestan University, Khoramabad, Iran

[7]Department of Physical Education and Sport Sciences, Faculty of Literature and Humanities, Vali-E-Asr University of Rafsanjan, Rafsanjan, Iran

[8]Research and Development Unit, Parc Sanitari Sant Joan de Deu, Barcelona, Spain

[9]Centre for Health, Performance and Wellbeing, Anglia Ruskin University, Cambridge, UK

[10]Department of Internal Medicine, Gachon University College of Medicine, Incheon, South Korea

[11]Division of Endocrinology and Metabolism, Department of Internal Medicine, Kyung Hee University Hospital at Gangdong and Kyung Hee University School of Medicine, Seoul, South Korea

[12]Division of Endocrinology and Metabolism, Department of Internal Medicine, Research Institute of Clinical Medicine of Jeonbuk National University and Jeonbuk National University Hospital, Jeonju, South Korea

[13]Department of Medical Nutrition, Graduate School of East-West Medical Science, Kyung Hee University, Yongin, South Korea

[14]Department of Pediatrics, Kyung Hee University College of Medicine, Seoul, South Korea

[15]Department of Endocrinology and Metabolism, Kyung Hee University School of Medicine, Seoul, South Korea

[†]The authors contributed equally.

***Corresponding authors**

Sang Youl Rhee, MD, PhD

Department of Endocrinology and Metabolism

Kyung Hee University College of Medicine

23 Kyungheedae-ro, Dongdaemun-gu, Seoul 02447, South Korea

Phone: 82 2 958 8200

E-mail: rheesy@khu.ac.kr

Dong Keon Yon, MD, PhD, FACAAI, FAAAAI

Department of Pediatrics

Kyung Hee University College of Medicine

23 Kyungheedae-ro, Dongdaemun-gu, Seoul 02447, South Korea

Phone: 82 2 6935 2476

E-mail: yonkkang@gmail.com

Supplementary Table S1. Baseline characteristics for the range of each continuous variable in the study and extra-validation datasets.

	**Study dataset**			**Extra-validation dataset**		
	**Total**	**Control**	**Case[a]**	**Total**	**Control**	**Case[a]**
BMI range, kg/m^2, mean (SD)	1.05 (1.87)	1.09 (1.91)	0.67 (1.33)	1.36 (1.67)	1.39 (1.68)	0.57 (1.07)
SBP range, mmHg, mean (SD)	9.67 (14.67)	10.02 (14.87)	6.38 (12.09)	19.26 (16.55)	19.56 (16.57)	8.43 (11.40)
DBP range, mmHg, mean (SD)	6.12 (9.40)	6.35 (9.55)	3.93 (7.55)	12.55 (10.32)	12.72 (10.30)	6.26 (8.81)
PR range, bpm, mean (SD)	6.86 (10.06)	7.12 (10.21)	4.39 (8.13)	13.58 (11.28)	13.68 (11.30)	9.62 (10.04)
HbA1c range, %, mean (SD)	1.22 (1.20)	1.30 (1.21)	0.39 (0.76)	1.35 (1.23)	1.37 (1.23)	0.54 (0.78)
Glucose range, mg/dL, mean (SD)	73.20 (72.24)	77.86 (72.67)	29.59 (50.41)	48.88 (62.98)	49.17 (63.07)	38.32 (59.27)
TC range, mg/dL, mean (SD)	47.63 (41.87)	50.61 (41.82)	19.72 (30.55)	48.81 (39.54)	49.35 (39.51)	28.64 (35.33)
TG range, mg/dL, mean (SD)	86.18 (88.37)	92.11 (89.11)	30.82 (56.62)	82.78 (80.73)	83.91 (80.98)	40.62 (56.72)
HDL-c range, mg/dL, mean (SD)	11.93 (13.03)	12.72 (13.05)	4.58 (10.32)	9.87 (9.48)	10.01 (9.51)	4.61 (6.86)
LDL-c range, mg/dL, mean (SD)	32.74 (33.84)	34.96 (34.09)	12.06 (22.61)	23.81 (31.37)	24.06 (31.45)	14.62 (26.81)
Creatinine range, mg/dL, mean (SD)	0.30 (0.45)	0.32 (0.46)	0.10 (0.22)	0.27 (0.59)	0.27 (0.60)	0.11 (0.19)
AST range, U/L, mean (SD)	17.70 (32.82)	19.03 (33.92)	5.25 (14.75)	10.27 (11.39)	10.43 (11.45)	4.13 (6.41)
ALT range, U/L, mean (SD)	18.77 (22.60)	20.11 (23.08)	6.29 (11.54)	14.02 (14.74)	14.22 (14.78)	6.45 (10.52)
GGT range, U/L, mean (SD)	27.12 (45.03)	28.90 (46.20)	10.47 (26.93)	18.80 (30.34)	19.05 (30.42)	9.30 (25.54)
ALP range, U/L, mean (SD)	28.37 (30.38)	30.32 (30.82)	10.11 (17.42)	37.55 (47.66)	38.10 (47.63)	17.04 (44.57)

[a]The group of the patients with newly outbreak of cardiovascular disease within 3 years.

BMI, body mass index; SD, standard deviation; SBP, systolic blood pressure; DBP, diastolic blood pressure; PR, pulse rate; HbA1c, glycated hemoglobin; TC, total cholesterol; TG: triglyceride; HDL-c, high-density lipoprotein cholesterol; LDL-c, low-density lipoprotein cholesterol; AST, aspartate aminotransferase; ALT, alanine aminotransferase; GGT, gamma-glutamyl transferase; ALP, alkaline phosphatase

Supplementary Table S2. Hyperparameters of the models.

Models	Hyperparameter
XGB	booster: gbtree, n_estimators: 100, max_depth: 19, learning_rate: 0.07, subsample: 0.4, eval_metric: logloss, scale_pos_weight: 9, tree_method: hist
RF	n_estimators: 120, max_depth: 8, max_features: sqrt
LGM	n_estimators: 130, learning_rate: 0.2, max_depth: 4, num_leaves: 200, subsample: 0.3, colsample_bytree: 1.0, objective: binary, n_jobs: -1
ADB	n_estimators: 50, learning_rate: 1, estimator: DecisionTreeClassifier(max_depth=2)
LR	penalty: l2, C: 0.233572146909012, solver: liblinear, max_iter: 10000
SVM	kernel: linear, probability: True, max_iter: 10000

XGB, XGBoost; RF, random forest; LGM, LightGBM; ADB, AdaBoost; LR, logistic regression; SVM, support vector machine.

Supplementary Table S3. Process of hyperparameter tuning in the random forest model.

Hyperparameter	Dataset	AUROC
n_estimator = 30<br>max_depth = 3	Original dataset	0.806 (0.793–0.819)
	External dataset	0.735 (0.672–0.794)
n_estimator = 50<br>max_depth = 3	Original dataset	0.808 (0.795–0.821)
	External dataset	0.740 (0.677–0.798)
n_estimator = 100<br>max_depth = 3	Original dataset	0.810 (0.797–0.823)
	External dataset	0.745 (0.685–0.799)
n_estimator = 120<br>max_depth = 3	Original dataset	0.810 (0.797–0.822)
	External dataset	0.748 (0.691–0.802)
n_estimator = 250<br>max_depth = 3	Original dataset	0.810 (0.797–0.822)
	External dataset	0.748 (0.690–0.802)
n_estimator = 120<br>max_depth = 4	Original dataset	0.816 (0.803–0.828)
	External dataset	0.742 (0.683–0.801)
n_estimator = 120<br>max_depth = 5	Original dataset	0.821 (0.808–0.834)
	External dataset	0.736 (0.679–0.793)
n_estimator = 120<br>max_depth = 7	Original dataset	0.827 (0.814–0.839)
	External dataset	0.726 (0.664–0.786)
n_estimator = 120<br>max_depth = 8	Original dataset	0.830 (0.818–0.842)
	External dataset	0.722 (0.660–0.783)

This process shows the tuning process of the random forest which was selected for having the best AUROC at validation dataset.
AUROC, area under the receiver operating characteristic curve.

Supplementary Fig. S1. Distribution of top 15 feature importance.

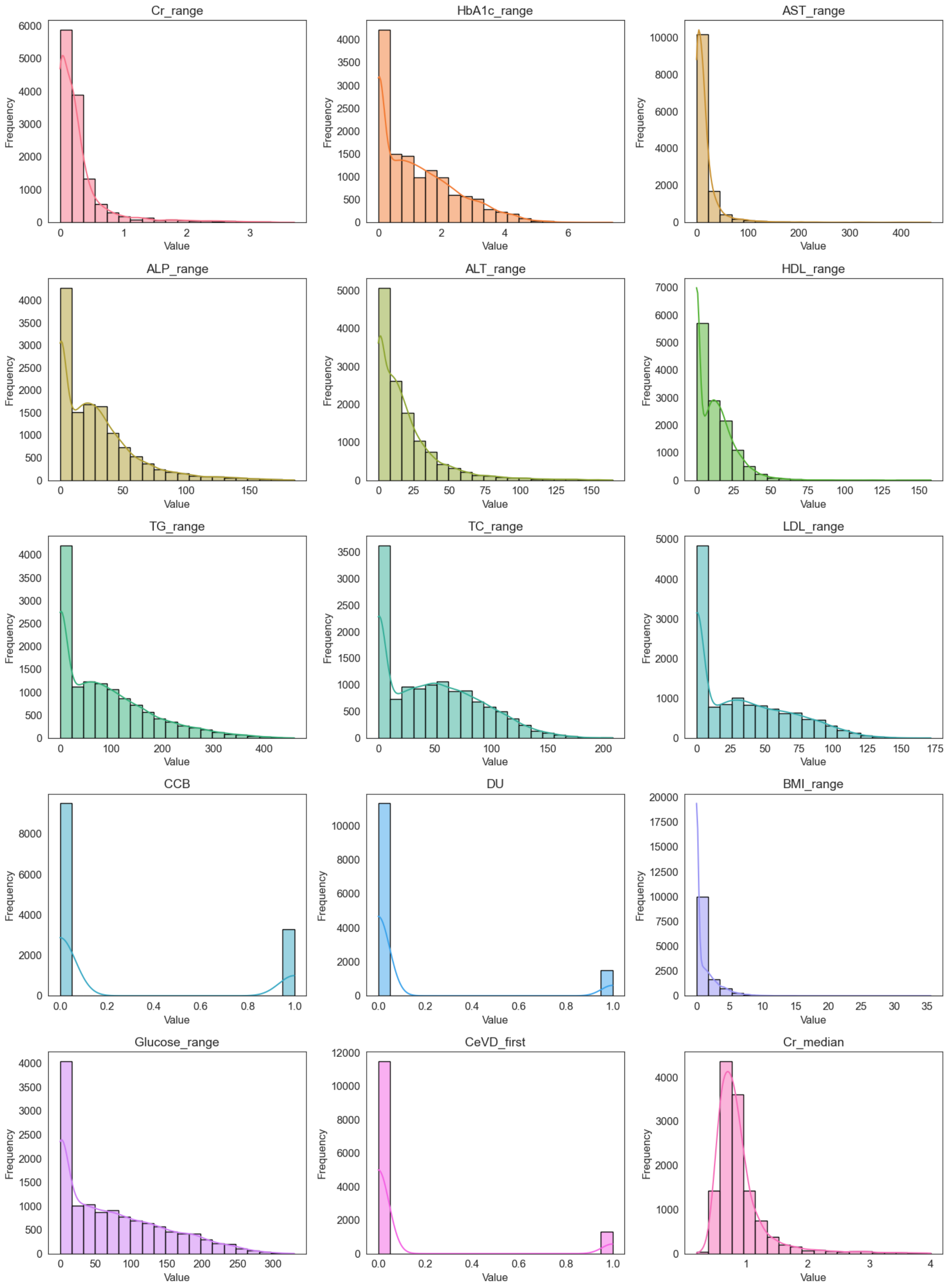

Supplement: Supplementary file 1 — Supplementary Information. [file 41598_2024_63798_MOESM1_ESM.pdf]
